# Accuracy of digital dental models and three-dimensional printed dental models in linear measurements and Bolton analysis

**DOI:** 10.12688/f1000research.31865.2

**Published:** 2021-09-01

**Authors:** William Suryajaya, Maria Purbiati, Nada Ismah

**Affiliations:** 1 Department of Orthodontics, Faculty of Dentistry, University of Indonesia, Jakarta, Indonesia

**Keywords:** Accuracy, 3D printing, digital dental model, printed dental model

## Abstract

**Background:** Due to advances in digital technology, it is possible to obtain digital dental models through intraoral scanning. The stereolithographic data collected from the scanner can subsequently be printed into a three-dimensional dental model in resinic material. However, the accuracy between digital dental models and printed dental models needs to be evaluated since it might affect diagnosis and treatment planning in orthodontic treatment.

This study aimed to evaluate the accuracy of digital models scanned by a Trios intraoral scanner and three-dimensional dental models printed using a Formlabs 2 3D printer in linear measurements and Bolton analysis.

**Methods: **A total of 35 subjects were included in this study. All subjects were scanned using a Trios intraoral scanner to obtain digital study models. Stereolithographic data from previous scanning was printed using a Formlabs 2 3D printer to obtain printed study models. Mesiodistal, intercanine, intermolar, and Bolton analysis from all types of study models were measured. The intraclass correlation coefficient was used to assess intraobserver and interobserver reliability. All data were then statistically analyzed.

**Results: **The reliability tests were high for both intraobserver and interobserver reliability, which demonstrates high reproducibility for all measurements on all model types. Most of the data compared between study models showed no statistically significant differences, though some data differed significantly. However, the differences are considered clinically insignificant.

**Conclusion: **Digital dental models and three-dimensional printed dental models may be used interchangeably with plaster dental models for diagnostic and treatment planning purposes.

**Keywords: **Accuracy, 3D printing, digital dental model, printed dental model.

## Introduction

Dental models are essential in the process of determining diagnosis and treatment planning in orthodontic treatment ^1– 3^. Commonly used dental models are made of plaster, which can easily be fractured and lost ^4^. Aside from that, plaster models require storage room, which can be problematic since typical dental practices have limited space, and the number of models will continue to grow as the number of patients treated grows ^5^. Moreover, the process of obtaining plaster models requires taking impressions with impression material, which can be an unpleasant experience for patients ^6^.

Recent developments in digital technology have made digitalization of dental models possible. Using an intraoral scanner, a patient’s oral condition, especially the teeth, can be registered and stored in a computer ^7^. The scanned data is in the form of stereolithographic data that can be retrieved from the computer storage system almost instantly. The advantage of digital study models is that they are not prone to damage, fracture, or loss. Not only is no extra space needed to store the models, digital models also make setting up models and sending models for referral easy ^8– 10^.

Generally, intraoral scanners record intraoral structures with a camera located on the tip of a wand that emits light. The light is then reflected back by the surface of the intraoral structures and recaptured by the camera to create digital objects ^11^. There are several intraoral scanners available on the market, such as iTero (Align Technologies, San Jose, CA), Lava Chairside Oral Scanner (COS) (3M ESPE, Seefeld, Germany), and Trios (3Shape, Copenhagen, Denmark). Of these scanners, Lava COS requires the addition of titanium oxide to opacify the tooth surface so that the light from the camera on the wand can be optimally reflected. This addition may affect the accuracy of the recorded digital model since it adds thickness to the teeth ^12^.

Stereolithographic data obtained from previous scanning can be printed using a 3D printer to produce a printed model ^8, 9^. When necessary, the printed model is especially beneficial in diagnosing complex cases when a tangible model would make the diagnosis easier. There are several 3D printer technologies available, such as Fusion Deposition Modelling, an inkjet-based system or 3DP, and stereolithography (SLA). Each 3D printer has its own method of producing 3D objects ^13^. However, the accuracy of a printed model may be altered since two steps are required to produce the printed model: scanning from the intraoral structure and printing it into resinic material.

The accuracy of dental models in recording intraoral structures is paramount since inaccuracy may lead to inaccurate diagnosis and treatment planning ^14^. Hence, it is important to evaluate the accuracy of various dental models compared to the commonly accepted instrument: plaster models. Several studies have confirmed that digital models are suitable to replace plaster models ^11, 14, 15^. However, limited studies are available that assess the accuracy of printed models, which have to go through the two steps of scanning and printing. This study aims to evaluate the linear accuracy and Bolton analysis ^16^ of digital dental models scanned using Trios and resinic dental models printed using Formlabs 2 and compare them to plaster dental models.

## Methods

### Subjects

This prospective observational analytical study was approved by the Ethics Committee of the University of Indonesia (approval number of 49/Ethics Approval/FKG UI/VI/2019). The subjects were graduate and undergraduate dental students at the University of Indonesia who agreed to participate in the study and signed informed consents after being briefed about the details of the study. Sample size was calculated using Gpower Software version 3.1 for windows with the premises: normal data distribution, α = 0.05, β = 80%, and effect size = 0.5 and the result signified a minimum sample of 34. Based on convenience sampling, a total of 35 subjects (mean age: 24.85 ± 3.9 years old), five males and 30 females, were selected based on the following inclusion criteria: (1) 16–50 years old, (2) total crowding on each jaw not exceeding 3 mm, and (3) all teeth from central incisors to first molars on each quadrant are present. The exclusion criteria were: (1) large filling or restoration on the proximal side of the measured teeth and (2) incomplete impression or scanning results. This study was conducted at Graduate Orthodontic Clinic at the University of Indonesia Teaching Dental Hospital between June 2019 until August 2019.

### Digital model

All subjects were scanned using Trios (3Shape, Copenhagen, Denmark) on both the maxilla and mandible. All teeth from the central incisors to the first molars were thoroughly scanned so as to produce complete sets of teeth on the digital model. Stereolithographic data from the scanning procedure were saved on a computer hard drive.

### Printed model

All stereolithographic data were subsequently printed using a Formlabs 2 SLA 3D printer (Formlabs, Somerville, MA) to produce printed dental models of both the upper and lower jaws of all subjects.

### Plaster model

After the scanning procedure was complete, impressions were taken from all subjects with alginate impression material (Hydrogum, Zhermack Dental, Badia Polesine, Italy). The impression was consequently poured with type II dental stone (Pro Model Super 11, Saint Gobain, France) to obtain a plaster dental model.

### Data collection

The mesiodistal widths of all teeth from the central incisors to the first molars on each quadrant of all models were measured. Subsequently, the intercanine and intermolar widths were also measured. All measurements on the plaster and printed models were measured using a digital caliper (Mitutoyo, Japan), while the digital models were measured using built-in measurement tools on Autodesk Netfabb Premium 2019 (RRID:SCR_019812) software as described in previous study by Akyalcin *et al*. ^17^ Approximately 10% of each model was measured by another observer (MP) to assess interobserver reliability. Interobserver reliability was assessed using the intraclass coefficient (ICC) and showed almost perfect agreement (ICC > 0.9). Within two weeks after the first measurements, 10% of all models were measured again by the same observer (WS) to assess intraobserver reliability. Intraobserver reliability was assessed using the ICC and reached almost perfect agreement (ICC > 0.9) between the first and second observations. Bolton analysis was then measured using the collected mesiodistal width data.

### Statistical analysis

All data were analyzed using the Statistical Package for the Social Sciences (SPSS) version 20.0 for Windows (RRID:SCR_019096). The data normality of each group was assessed using the Shapiro-Wilk test. Repeated measures ANOVA was used for parametric numeric data, while the Friedman test was used to compare nonparametric numeric data. Within-group differences were assessed using the paired t-test for parametric data sets, while the Wilcoxon test was used for nonparametric data sets. A p-value < 0.05 was considered statistically significant.

## Results

**Figure 1.**
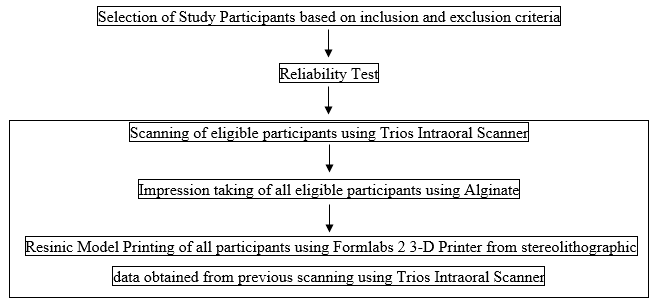
Flow of Events.

The flow of events is shown in Figure 1.

### Reliability test

The reliability of all criteria in this study was tested using the ICC. All data tested, including mesiodistal, intercanine, and intermolar width for both interobserver and intraobserver, were found to be reliable with high agreement ( Table 1 and Table 2).

**Table 1.** Intraobserver reliability.

Component	Agreement Intraobserver *		
	Plaster Model	Digital Model	Resin Model
Mesiodistal	0.991	0.989	0.993
Intercanine	0.994	0.969	0.999
Intermolar	0.999	0.998	0.998

*: ICC: <0.20: *poor agreement* | 0.21–0.40: *fair agreement* | 0.41–0.60: *moderate agreem*ent | 0.61–0.80: *substantial agreement* | 0.81–1.00: *almost perfect agreement*

**Table 2.** Interobserver reliability.

Component	Agreement Interobserver *		
	Plaster Model	Digital Model	Resin Model
Mesiodistal	0.991	0.958	0.990
Intercanine	0.994	0.997	0.993
Intermolar	0.988	0.997	0.993

*: ICC: <0.20: *poor agreement* | 0.21–0.40: *fair agreement* | 0.41–0.60: *moderate agreem*ent | 0.61–0.80: *substantial agreement* | 0.81–1.00: *almost perfect agreement*

### Mesiodistal width

**Graph 1.**
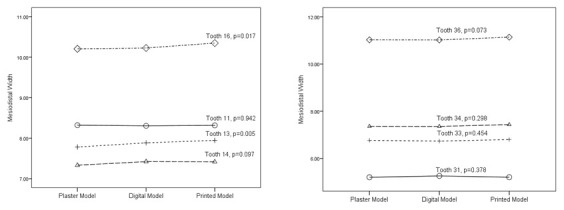
Mesiodistal Width Mean Comparison.

A total of eight different teeth (11, 13, 14, 16, 31, 33, 34, 36) were chosen to represent each of the tooth types on each jaw. Almost all data were normally distributed except 11 and 31. This is probably due to the various tooth sizes between individual subjects. All group comparisons ( Graph 1) showed statistically significant differences (p<0.05) on 13 and 16. Further within-group analysis ( Graph 1) revealed statistically significant differences (p<0.05) between printed models and plaster models on both teeth.

### Intercanine and intermolar width

**Graph 2.**
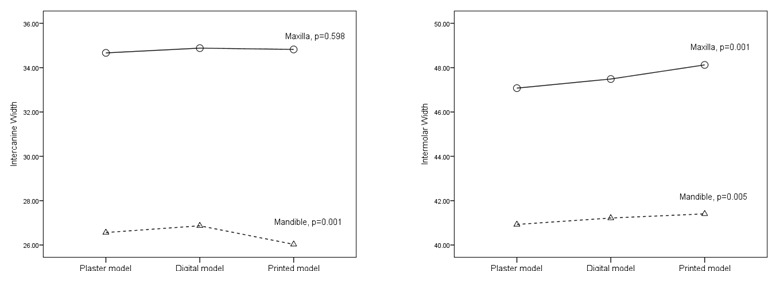
Mesiodistal Width Mean Comparison.

The intercanine and intermolar widths of all groups were compared, and significant differences (p<0.05) were found for mandibular intercanine, maxillary intermolar, and mandibular intermolar ( Graph 2). Further comparison analysis between groups revealed a significant difference for maxillary intercanine between the digital model-printed model and printed model-plaster model ( Graph 2). Significant differences (p<0.05) between groups were found for the digital model-printed model and the printed model-plaster model for the maxillary intermolar teeth and for the printed model-plaster model and plaster model-digital model for mandibular intermolar teeth.

### Bolton analysis

**Graph 3.**
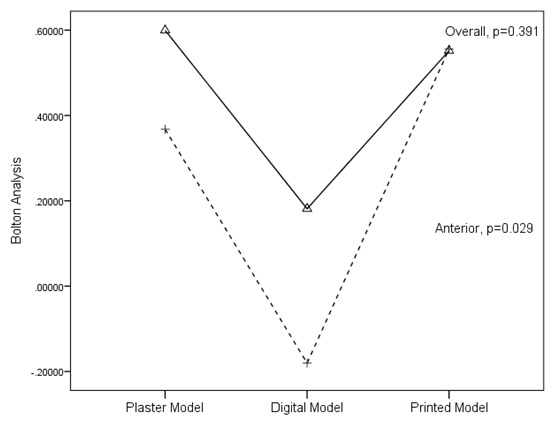
Bolton Analysis Mean Comparison.

The data collected for the Bolton analysis were compared. A significant difference (p<0.05) was found on anterior Bolton analysis ( Graph 3). A positive result showed tooth excess on maxillary teeth, and a negative result showed tooth excess on mandibular teeth. Comparisons between groups showed significant differences for the printed model-plaster model and the plaster model-digital model on anterior Bolton analysis ( Graph 3).

## Discussion

Mesiodistal width, intercanine width, intermolar width, and Bolton analysis were compared between the plaster models, digital models, and printed models. Measurements on the digital models were found to be challenging, as a digital model is a three-dimensional object to be seen on the two-dimensional spectrum of a computer screen. All measurements were conducted by one observer, which makes the measurements more reliable ^8^.

While the comparison of the mesiodistal widths of the measured teeth showed varied results, most of the teeth measured had no statistically significant difference. If the difference was found to be significant, it was within the range of 0.15 mm, which was deemed clinically insignificant. It must be noted that between-group comparison revealed the difference was between the plaster models and printed models. The same result was found by Brown *et al*. ^18^ The difference is probably due to instability of alginate impressions. Alginate is unstable at room temperature, so it can absorb water from the air through the process of imbibition, which causes alginate to enlarge ^19^. Moreover, 3D printer minimum thickness may play a role in the difference. The minimum thickness can cause the printed model to be bigger than usual even though the minimum thickness is relatively small (25 μm). The differences for mesiodistal width between the plaster models and digital models were found to be insignificant.

Intercanine and intermolar comparison in this study showed statistically significant differences for mandibular intercanine, maxillary intermolar, and mandibular intermolar. However, even though the differences were statistically significant, they were rendered clinically insignificant since the mean difference was not more than 1.5 mm. De Waard *et al*. ^20^ found a similar result, although the digital models in their study were obtained from cone-beam computed tomography scanning of plaster models. Another study by Brown *et al*. ^18^ found that there were no statistically significant differences between printed models and plaster models. However, the 3D printer used in that study was a polyjet and DLP printer, which has a minimum thickness ranging from 15 μm to 50 μm, while the printer used in the present study was an SLA printer, which has a minimum thickness of 50 μm.

Bolton analysis measurement showed a statistically significant difference for anterior Bolton analysis. Even though the difference was significant, the Bolton analysis measurement difference was not more than 1.5 mm, which is clinically insignificant ^21^. Several studies have revealed the same result as this study for Bolton analysis comparison between different dental models ^8, 11^. Bolton analysis is a very sensitive technique. Measurement by the same observer on the same model may produce a different result ^22^. Hence, the statistically significant difference in this study was rendered clinically insignificant.

Several limitations and difficulties were present on this study. Plaster model as gold standard reference measurement does not represent the actual size of each tooth since both the impression material and the plaster used in the making of plaster model may shrink and cause disparity from actual tooth size. Measurement on dry skull as reference might be more appropriate on similar future study since measurement on patient is both difficult and inconvenience. Moreover, selection of 3D printer model might enhance the accuracy of the study. 3D printer that can print 3D object with less minimum thickness than that of used in this study is preferable to produce more accurate resin model.

## Conclusion

Digital models and printed models are suitable for diagnosing and treatment planning in orthodontic cases because the linear measurement and Bolton analysis between these different study models mostly showed no statistically significant differences. Even when there were statistically significant differences, it was negligible clinically. Moreover, in fact, nowadays, digital and printed model has been used by orthodontist worldwide to diagnose and to make treatment plan.

## Consent

All participants provided written informed consent before involvement in the study.

## Data availability

### Underlying data

Figshare: Research Data, https://doi.org/10.6084/m9.figshare.13469160.v1 ^23^

This project contains the following underlying data:

- Linear measurements
- Bolton Analysis of dental stone, digital, and printed models

Figshare: Stereolihographic Data, https://doi.org/10.6084/m9.figshare.13469172.v1 ^24^

This project contains the following underlying data:

- Stereolithographic data obtained from scanning subjects’ dentition.

Data are available under the terms of the Creative Commons Zero "No rights reserved" data waiver (CC0 1.0 Public domain dedication).
