# Supplementary material for: Accuracy of digital dental models and three-dimensional printed dental models in linear measurements and Bolton analysis
Source: F1000Res. 2021 Sep 1;10:180. Originally published 2021 Mar 4. [Version 2] doi: 10.12688/f1000research.31865.2 (PMC8078212; doi:10.12688/f1000research.31865.2)
Supplement: Peer review history

# Peer review: Reviewer response for version 2

**DOI:** 10.5256/f1000research.76989.r93200

**Published:** 2021-09-13

**Authors:** Cortino Sukotjo

**Affiliations:** 1 Department of Restorative Dentistry, University of Illinois at Chicago, Chicago, IL, USA

Thank you for revising the manuscript.

Is the work clearly and accurately presented and does it cite the current literature?

Partly

If applicable, is the statistical analysis and its interpretation appropriate?

Yes

Are all the source data underlying the results available to ensure full reproducibility?

Yes

Is the study design appropriate and is the work technically sound?

Partly

Are the conclusions drawn adequately supported by the results?

No

Are sufficient details of methods and analysis provided to allow replication by others?

Yes

Reviewer Expertise:

Research

I confirm that I have read this submission and believe that I have an appropriate level of expertise to confirm that it is of an acceptable scientific standard.

# Peer review: Reviewer response for version 1

**DOI:** 10.5256/f1000research.34922.r80963

**Published:** 2021-04-26

**Authors:** Ashok Karad

**Affiliations:** 1 Smile Care, Mumbai, Maharashtra, India

Dental study models are one of the important diagnostic aids and their accuracy affects the diagnosis and treatment planning. This study has evaluated the linear accuracy and Bolton analysis of digital dental models and printed models and compared them to plaster dental models. The authors have done good work in analysing various parameters and providing a useful and clinically-relevant information. The contents have been clearly and accurately presented in the manuscript. The results of the study have been discussed comprehensively and are supported with appropriate references.

 However, the following minor concerns need to be addressed by the authors to provide more clarity and better understanding:

1. The manuscript requires one more round of linguistic/ grammatical editing.
2. In ‘Methods’ section, it is appropriate to use the term ‘Ethics committee’ instead of ‘Ethical committee’.
3. The figure 1 legend should be ‘Flow of events’ instead of ‘Flow of participants’. Make corresponding changes in the manuscript as well.

Is the work clearly and accurately presented and does it cite the current literature?

Yes

If applicable, is the statistical analysis and its interpretation appropriate?

I cannot comment. A qualified statistician is required.

Are all the source data underlying the results available to ensure full reproducibility?

Yes

Is the study design appropriate and is the work technically sound?

Yes

Are the conclusions drawn adequately supported by the results?

Yes

Are sufficient details of methods and analysis provided to allow replication by others?

Yes

Reviewer Expertise:

Orthodontic clinical work and research

I confirm that I have read this submission and believe that I have an appropriate level of expertise to confirm that it is of an acceptable scientific standard.

# Response

**Published:** 2021-08-24

**Authors:** William Suryajaya

**Affiliations:** University of Indonesia, Indonesia

Dear Reviewer,

 Thank you for your valuable inputs. Here are our response:

 1. We have made an attempt to cross this article through a professional grammar editing process.

 2. Noted and will be revised.

 3. Noted and will be revised.

 Thank you

# Peer review: Reviewer response for version 1

**DOI:** 10.5256/f1000research.34922.r80964

**Published:** 2021-03-29

**Authors:** Cortino Sukotjo

**Affiliations:** 1 Department of Restorative Dentistry, University of Illinois at Chicago, Chicago, IL, USA

Dear authors, thank you very much for your article.

1. Please convert table 3-9 to graphs, so that you can include p-value comparisons between group. 
2. The gold standard is plaster model, however, you mentioned many disadvantages of plaster model, which could confuse the reader who is not orthodontist.
3. You mentioned that some values were significantly different, but were they clinically significant?
4. In your conclusion, you mentioned that: *"Digital models and printed models may be considered to replace plaster models to diagnose and plan treatment for orthodontic cases"*. Actually, in many developed countries, digital models have been used in orthodontic offices daily. Please revise your conclusion.

Is the work clearly and accurately presented and does it cite the current literature?

Partly

If applicable, is the statistical analysis and its interpretation appropriate?

Yes

Are all the source data underlying the results available to ensure full reproducibility?

Yes

Is the study design appropriate and is the work technically sound?

Partly

Are the conclusions drawn adequately supported by the results?

No

Are sufficient details of methods and analysis provided to allow replication by others?

Yes

Reviewer Expertise:

Research

I confirm that I have read this submission and believe that I have an appropriate level of expertise to confirm that it is of an acceptable scientific standard, however I have significant reservations, as outlined above.

# Response

**Published:** 2021-08-24

**Authors:** William Suryajaya

**Affiliations:** University of Indonesia, Indonesia

Dear reviewer,

 Thank you for the valuable input and here are our response:

 1. Yes, we have replaced the tables with graphs so as to make the reader easier to compare between the p-values.

 2. Plaster model is still the gold-standard study models in most parts of the worlds, especially in the less developed country. However, since it has many disadvantages, efforts to replace them with a more precise and accurate models has been very rigorous. One of the effort is to replace them with digital models and printed models.

 3. As mentioned, a couple of times in the article and citing some references, we mentioned that the differences are clinically insignificant though statistically significant.

 4. We have made revision accordingly.

 Thank you.

# Peer review: Reviewer response for version 1

**DOI:** 10.5256/f1000research.34922.r80961

**Published:** 2021-03-17

**Authors:** Ida Bagus Narmada

**Affiliations:** 1 Department of Orthodontics, Faculty of Dental Medicine, Airlangga University, Surabaya, Indonesia

The authors of this study have evaluated the linear accuracy and Bolton analysis of digital dental models scanned using Trios and resinic dental models printed using Formlabs 2 and compare them to plaster dental models. The results are certainly interesting and the authors are commended for executing this study. 

 However, the authors need to address the following minor concerns: 

**General**:

- The paper is well organized and easy to follow.
- Novelty is sufficient and high impact.
- ​​​​​To improve the readability, it is recommended that the text is checked by a native English speaking person as many of the sentences might be misunderstood. I suggest a revision of the English grammar structures by an expert editor in revising manuscripts.

**Introduction**:

- No revision is needed.

**Results:**

- Please provide and add some information about standard deviation of the data.
- Is it possible to combine the tables to reduce the number of the tables? (9 tables are too many).

**Discussion**:

- Discussion of the results is quite comprehensive. In analyzing the results, the authors also show citations from the previous study to support the explanation of these results.
- The answer to the hypothesis of this study should be included at the beginning of the discussion section.
- Please mention the limitation of this study and the suggestion for future study in the discussion section.

**Conclusion:**

- The conclusion section is quite comprehensive. However, please add the clinical implication from this study result.

**References**:

- The supporting references are adequate for the medium article.

Is the work clearly and accurately presented and does it cite the current literature?

Yes

If applicable, is the statistical analysis and its interpretation appropriate?

Partly

Are all the source data underlying the results available to ensure full reproducibility?

Partly

Is the study design appropriate and is the work technically sound?

Yes

Are the conclusions drawn adequately supported by the results?

Yes

Are sufficient details of methods and analysis provided to allow replication by others?

Yes

Reviewer Expertise:

Orthodontics and Orthopaedic Dentofacial

I confirm that I have read this submission and believe that I have an appropriate level of expertise to confirm that it is of an acceptable scientific standard.

# Response

**Published:** 2021-08-24

**Authors:** William Suryajaya

**Affiliations:** University of Indonesia, Indonesia

Dear Reviewer,

 Thank you for the valuable suggestions. Here are our response:

 We have made revisions accordingly. Notably, We replaced the tables with graphs so as to limit the number of the tables in the article.

 Thank you
